# The association between the change in severity score from baseline and the outcomes of critically ill patients was enhanced by integration of bioimpedance analysis parameters

**DOI:** 10.1038/s41598-024-65782-y

**Published:** 2024-06-25

**Authors:** Zhen Hu, Chuan Li, Shuhua Zhu, Yongchun Ge, Dehua Gong

**Affiliations:** National Clinical Research Center for Kidney Diseases, Jinling Hospital, Affiliated Hospital of Medical School, Nanjing University, 305 Zhongshan East Road, Xuanwu District, Nanjing, Jiangsu Province China

**Keywords:** Bioimpedance analysis, Phase angle, Severity scores, Dynamic change, Outcomes, Medical research, Outcomes research

## Abstract

The study of the outcomes of critically ill patients has been a hard stuff in the field of intensive care. To explore the relationship between changes of severity scores, bioelectrical impedance analysis (BIA) and outcomes of critically ill patients, we enrolled patients (n = 206) admitted to intensive care unit (ICU) in Jinling Hospital from 2018 to 2021 with records of BIA on the days 1- and 3- ICU. Collected BIA and clinical data including simplified acute physiology score II (SAPS II) and sequential organ failure assessment. According to the baseline and change of severity scores or phase angle (PA) values, the patients were divided into: G–G, baseline good status, 3rd day unchanged; G–B, baseline good status, 3rd day deteriorated; B–G, baseline bad status, 3rd day improved; and B–B, baseline bad status, 3rd day unchanged. According to PA, the mortality of group G–G was 8.6%, and it was greater than 50% in group B–B for severity scores. The new score combining PA and severity scores established. Multivariate logistic regression analysis revealed that PA–SAPS II score was the only independent factor for 90-day mortality (*P* < 0.05). A linear correlation was found between mortality and PA–SAPS II score (prediction equation: $Y(\%)=16.97\times X-9.67$, R^2^ = 0.96, *P* < 0.05).

## Introduction

Various severity score systems, including the simplified acute physiology score II (SAPS II), sequential organ failure assessment (SOFA), and acute physiology and chronic health evaluation II (APACHE II), have been developed to assess disease severity and to predict the outcomes of critically ill patients^1–4^. The utility of these systems is usually based on static data obtained at a specific time-point^5–8^. An early study showed that the maximum SOFA score during the intensive care unit (ICU) stay is more effective than the initial score for evaluating prognosis^9^. The APACHE II score before discharge is better than that at admission for predicting the mortality of severely ill surgical patients^10^. Although continuous efforts have been made to improve the prediction accuracy of these scoring systems, some limits have been revealed by previous studies. For example, mortality may increase with increasing SAPS II score when it is at a high level but change little when it is < 30^11,12^, which suggests that low SAPS II scores are unsafe in these patients. However, for dynamic changes in scores, additional studies are needed to investigate the role of these scores in the evaluation of clinical conditions and outcomes.

Bioelectrical impedance analysis (BIA) is a noninvasive method for measuring the body’s electrical impedance at alternating current frequencies^13^. As one of the important indicators of BIA, the phase angle (PA) reflects the reactance of the body^14^ and has been found to be related to health and the prognosis of the disease^15–19^. Because BIA, a reflection of the body’s bioelectric characteristics, is quite different from severity scores originating from clinical conditions and laboratory test results, it is rational to expect a complementary effect by combining both methods in the assessment of disease severity and prediction of outcome. This study aimed to investigate the effect of combining dynamic changes in severity scores and PA on the outcome of critically ill patients.

## Patients and methods

### Study population

We searched the hospital’s electronic medical records between June 2018 and June 2021 to enroll patients who stayed in the ICU of Jinling Hospital, were aged > 18 years, and had two BIA measurements (on admission to the ICU and the 3rd day). No pregnant women or patients with burns, prostheses, amputations, a cardiac pacemaker or a defibrillator were enrolled because BIA was not suitable for inclusion.

### BIA measurement and severity scores

The BIA measurements performed on admission to the ICU and on the 3rd day were collected, as were the SAPS II, SOFA and APACHE II scores. A Bodystat QuadScan 4000 device (Bodystat Ltd., Isle of Man, UK) was used at frequencies of 5, 50, 100, and 200 kHz for BIA. In accordance with the manufacturer’s instructions, a tetrapolar wrist-to-ankle method was used, in which two electrodes were placed approximately 5 mm apart on the dorsal surface of the right wrist and ipsilateral ankle with the patient in the supine position and with the arms and legs in abduction to avoid contact with the trunk. The input variables included the patients’ age, sex, height and actual body weight. The output parameters included PA (50 kHz), the ratio of impedance (IR) at high to low frequencies (200 kHz/5 kHz, IR), extracellular water (ECW), intracellular water (ICW) and total body water (TBW). TBW, ICW and ECW were calculated based on a built-in undisclosed proprietary equation developed by the manufacturer.

### Grouping of patients

According to the baseline and dynamic changes in severity scores or PAs, the patients were divided into 4 groups: (1) G–G, baseline good status, the 3rd day unchanged; (2) G–B, baseline good status, the 3rd day deteriorated; (3) B–G, baseline bad status, the 3rd day improved; and (4) B–B, baseline bad status, the 3rd day unchanged.

The baseline status was determined to be good (G) or bad (B) by a cutoff point obtained from the baseline severity scores or PA values using X-Tile software. The 3rd day status was determined according to the variation amplitude of severity scores or PA values from baseline to the 3rd day: the variation amplitude within the 0–25th percentile was considered to be improved, that within the 25–75th percentile was unchanged, and that within the 75–100th percentile was considered to be deteriorated.

### Ethics statement

The study protocol was previously approved by the Institutional Review Board of Jinling Hospital (2023DZKY-114-01). The requirement for informed consent was waived due to the retrospective design of the study. The study conforms to the principles outlined in the Declaration of Helsinki. All methods were carried out in accordance with relevant guidelines and regulations.

### Statistical analysis

Statistical analyses were performed using SPSS version 26.0 (IBM Corp., Armonk, NY) and GraphPad Prism 6.01 (GraphPad Software, Inc.) The normality of the data was tested by the Shapiro‒Wilk test. Normally distributed data are expressed as the mean ± standard deviation, and the rest are expressed as the median (interquartile range). For comparisons of survivors and non-survivors, an independent sample *t*-test was used for normally distributed data and homogeneous variance, and the Mann‒Whitney *U* test was used for the remaining data. The cutoff values for PA and severity score were calculated according to X-Tile software. A Kaplan‒Meier (K–M) survival curve was generated for survival analysis. Univariate and multivariate logistic regression analyses with a forward stepwise approach were performed to investigate correlations between BIA data and severity scores with respect to their ability to predict mortality. The goodness of fit of the linear regression equation was compared with the R^2^. A two-tailed *P* < 0.05 was considered to indicate statistical significance.

## Results

A total of 206 patients were enrolled, including 141 males (68.4%) with a mean age of 48.4 ± 17.4 years and a 90-day mortality of 31.1%. The primary reasons for admission to the ICU included acute pancreatitis (38.8%), renal diseases (28.2%), gastrointestinal diseases (12.1%), pneumonia (4.3%), trauma (2.9%), and others (13.7%). There were 98 patients (47.6%) with multiple organ dysfunction, 52 patients (25.2%) with sepsis, and 119 patients (57.8%) receiving continuous renal replacement therapy (CRRT). The median length of ICU stay was 19.0 days (interquartile 10.0, 36.0 days), and the median APACHE II, SOFA and SAPS II scores were 11.0 (interquartile 8.0, 16.0), 5.0 (interquartile 3.0, 9.0) and 28.5 (interquartile 20.0, 39.3), respectively.

### Baseline characteristics of 90-day survivors and non-survivors

The baseline characteristics of the 90-day survivors and non-survivors are shown in Table 1. Compared to 90-day survivors, non-survivors were older, had a higher rate of receiving CRRT, and higher levels of N-terminal pro-brain natriuretic peptide (NT-pro BNP) and C-reactive protein (CRP); higher severity scores; higher IR values; but lower hemoglobin (Hb), hematocrit (Hct), platelet (PLT), and PA levels (*P* < 0.05).

**Table 1:** Baseline characteristics of 90-day survivors and non-survivors.

	Survivors (n = 142)	Non-survivors (n = 64)	*P* value
Age(year)	42.0 (32.5, 59.5)	55.0 (38.0, 65.0)	0.012
Female/male	45/97	20/44	0.869
CRRT/non-CRRT	74/68	45/19	0.003
Clinical indicators			
NT-pro BNP (ng/L)	157.8 (47.8, 600.3)	283.4 (67.2, 1253.0)	0.033
Hb(g/L)	90.0 (79.0, 111.5)	82.0 (74.0, 100.0)	0.004
Hct	0.28 (0.24, 0.34)	0.27 (0.23, 0.32)	0.001
WBC (*10^9^/L)	8.4 (6.0, 12.9)	8.5 (6.0, 13.7)	0.728
PLT (*10^9^/L)	196.0 (123.5, 267.5)	109.5.0 (66.0, 150.5)	< 0.001
BUN (mg/dL)	9.3 (3.4, 18.2)	11.3(2.2, 23.6)	0.836
Cr (umol/L)	124.1 (59.1, 306.3)	129.7 (58.0, 242.0)	0.529
Alb (g/L)	30.9 (27.9, 33.1)	30.8 (26.7, 33.6)	0.707
TBIL (umol/L)	10.0 (6.2, 24.7)	13.4 (8.1, 30.9)	0.130
CRP (mg/L)	68.4 (9.7, 159.1)	130.9 (38.0, 180.8)	0.005
Severity scores			
APACHE II	10.0 (7.0, 15.0)	15.0 (11.0, 18.0)	< 0.001
SAPS II	25.0 (17.0, 35.0)	39.0 (27.0, 45.0)	< 0.001
SOFA	4.0 (3.0, 7.0)	8.0 (5.0, 12.5)	< 0.001
BIA parameters			
TBW (L)	39.2 (34.2, 47.3)	45.0 (34.6, 52.3)	0.083
TBW/weight (%)	59.4 (52.1, 65.7)	62.1 (54.0, 72.7)	0.068
ECW (L)	20.0 ± 7.2	20.7 ± 5.3	0.518
ECW/weight (%)	27.7 (25.1, 30.4)	28.8 (25.9, 32.6)	0.058
ECW/TBW (%)	47.1 (45.8, 47.9)	47.3 (46.4, 48.0)	0.441
ICW (L)	18.4 (15.5, 22.8)	19.8 (15.1, 23.1)	0.584
ICW/weight (%)	27.8 (25.7, 30.5)	28.1 (25.1, 31.7)	0.755
ICW/TBW (%)	47.1 (43.4, 50.1)	47.2 (43.6, 49.7)	0.319
FFM%	66.8 (43.1, 73.9)	72.0 (53.9, 87.2)	0.319
PA	3.7° (2.9°, 4.8°)	2.9° (2.5°, 3.7°)	< 0.001
IR	0.87 (0.84, 0.90)	0.89 (0.87, 0.91)	0.001

NT-pro BNP, N-terminal pro-brain natriuretic peptide; Hb, hemoglobin; Hct, hematocrit; WBC, white blood cell; PLT, platelets; BUN, blood urea nitrogen; Cr, creatinine; Alb, albumin; TBIL, total bilirubin; CRP, C-reactive protein; APACHE II, acute physiology and chronic health evaluation II; SAPS II, simplified acute physiology score II; SOFA, sequential organ failure assessment; TBW, total body water; ECW, extracellular water; ICW, intracellular water; FFM%, fat free mass%; PA, phase angle; IR, impedance ratio.

### Outcome comparisons between groups divided by the change trends in severity scores or PA values

**Figure 1:**
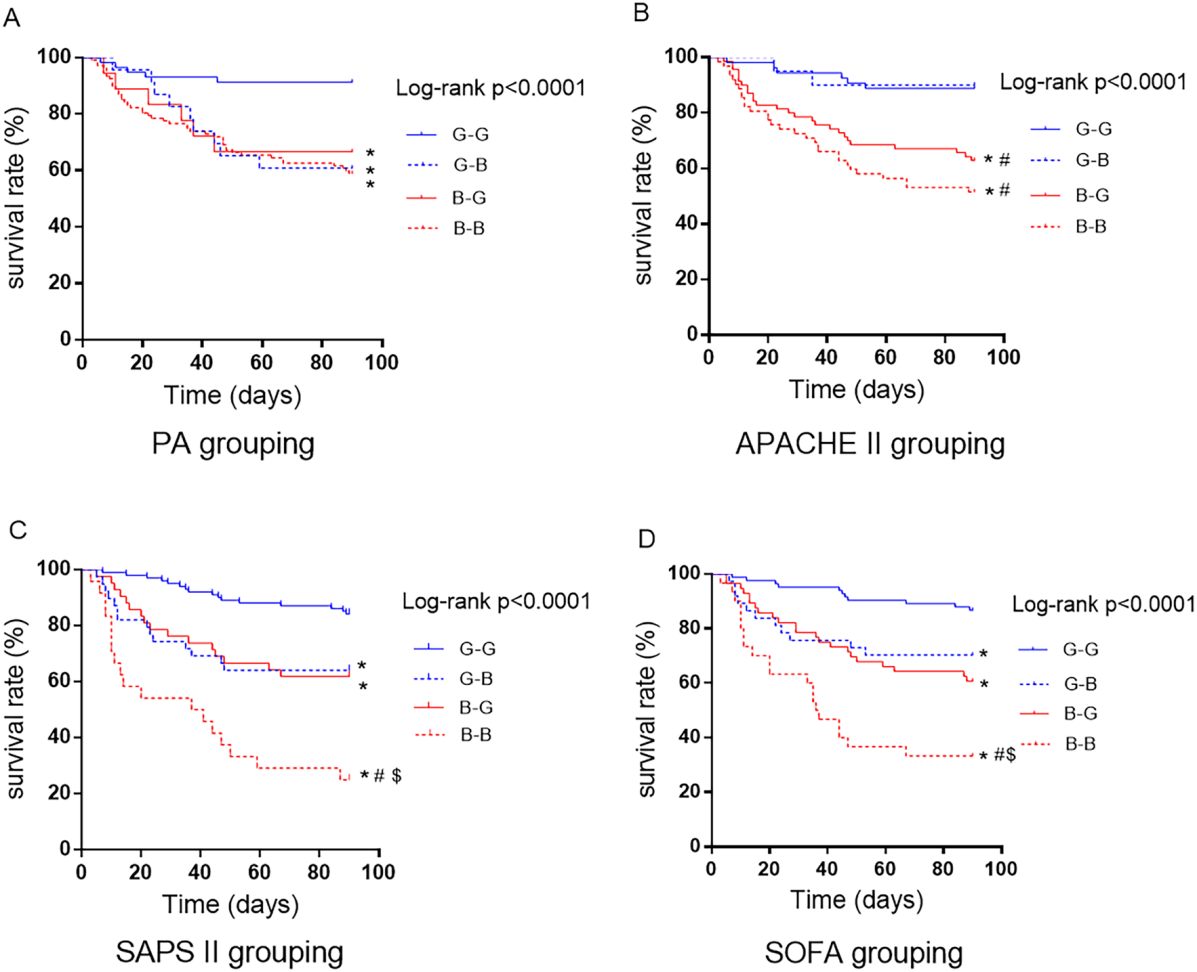
K‒M survival curves of patients grouped by change trends of indicators. The patients were grouped according to their baseline values and dynamic changes in severity scores or PA values: (1) G–G, baseline good status, unchanged on the 3rd day; (2) G–B, baseline good status, deteriorated on the 3rd day; (3) B–G, baseline bad status, improved on the 3rd day; and (4) B–B, baseline bad status, unchanged on the 3rd day. The baseline status was determined to be good (G) or bad (B) by the cutoff values obtained from the baseline severity scores or PA values using X-Tile software. The 3rd day status was determined according to the variation amplitude of severity scores or PA values from baseline to the 3rd day: the variation amplitude within the 0-25th percentile was considered to indicate improvement, that within the 25th–75th percentile was unchanged, and that within the 75th-100th percentile was considered to indicate deterioration. *, #, $, compared with Groups G–G, G–B, and B–G, respectively; *P* < 0.05.

As described above, the patients were divided into G–G, G–B, B–G, and B–B groups according to the change in severity score or PA value. K‒M survival analysis between the groups (Fig. 1) revealed significant differences in the cumulative survival rate between the groups stratified by PA and severity scores (*P* < 0.05). Interestingly, based on the PA values, the mortality of the G–G group was 8.6%, whereas that of the B–B group was 40.9%. For severity scores, the mortality rates were greater than 10% in group G–G (11.1%, 13.3%, and 15.8% based on the APACHE II score, SOFA score, and SAPS II score, respectively) and were close to or greater than 50% in group B–B (48.4%, 66.7%, and 76% for the APACHE II score, SOFA score and SAPS II score, respectively). The rate of receiving CRRT was 57.8% in the whole patients, and the highest rate was present in G–B group, regardless of grouping criteria (PA grouping: 73.4%, APACHE II grouping: 75.7%, SOFA grouping: 82.1%, SAPS II grouping: 76.2%). The difference of receiving CRRT rates between groups was not significant when grouping based on PA value or SASP II, but was significant when grouping based on APACHE II and SOFA.

### Combination of the PA value and severity score

**Figure 2:**
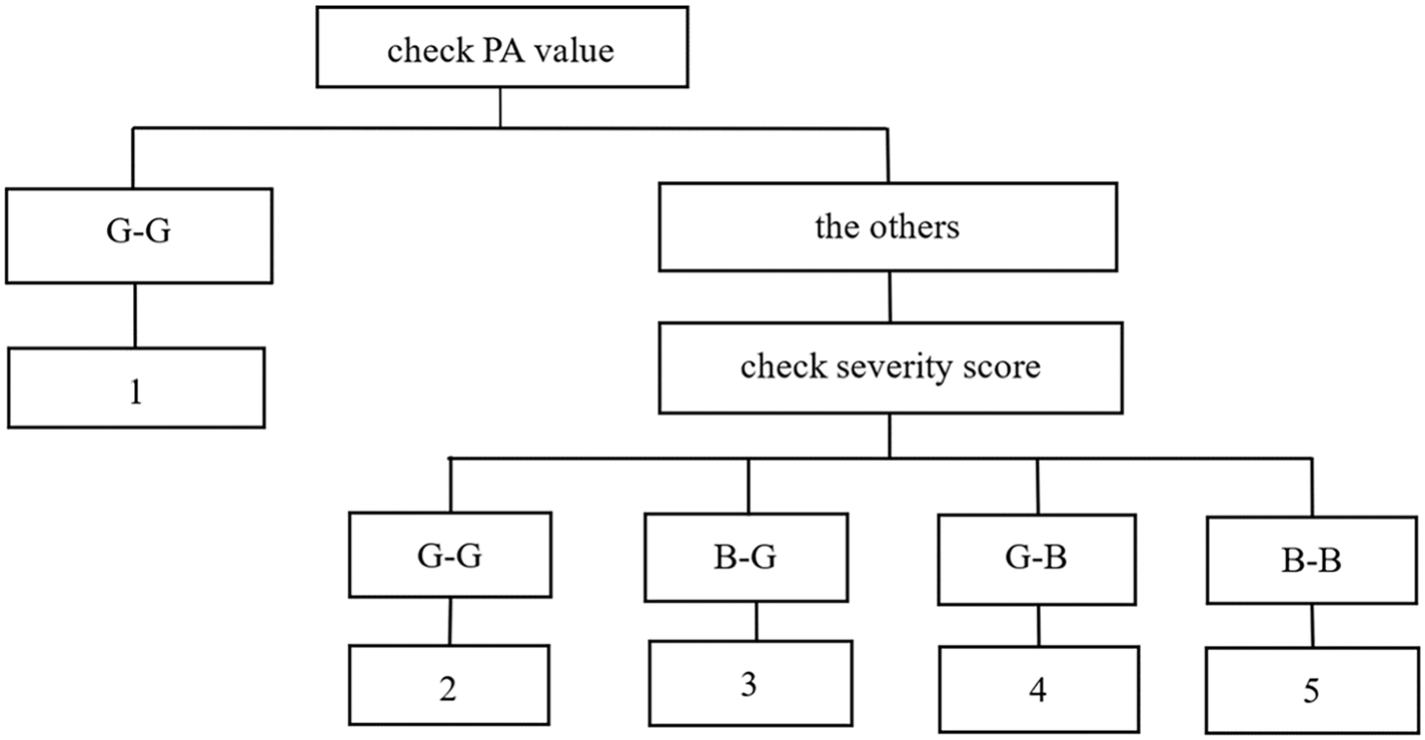
Flow diagram of the scoring process combining the PA and severity score systems. The severity score systems used included the APACHE II, SOFA and SAPS II.

To determine the ability of PA to predict lower mortality, we combined PA with 3 severity scores as follows: first, we checked the PA value to a score of G–G “1”, and second, we checked the severity score to a score of G–G “2”, B–G “3”, G–B “4”, or B–B “5” (Fig. 2).

**Figure 3:**
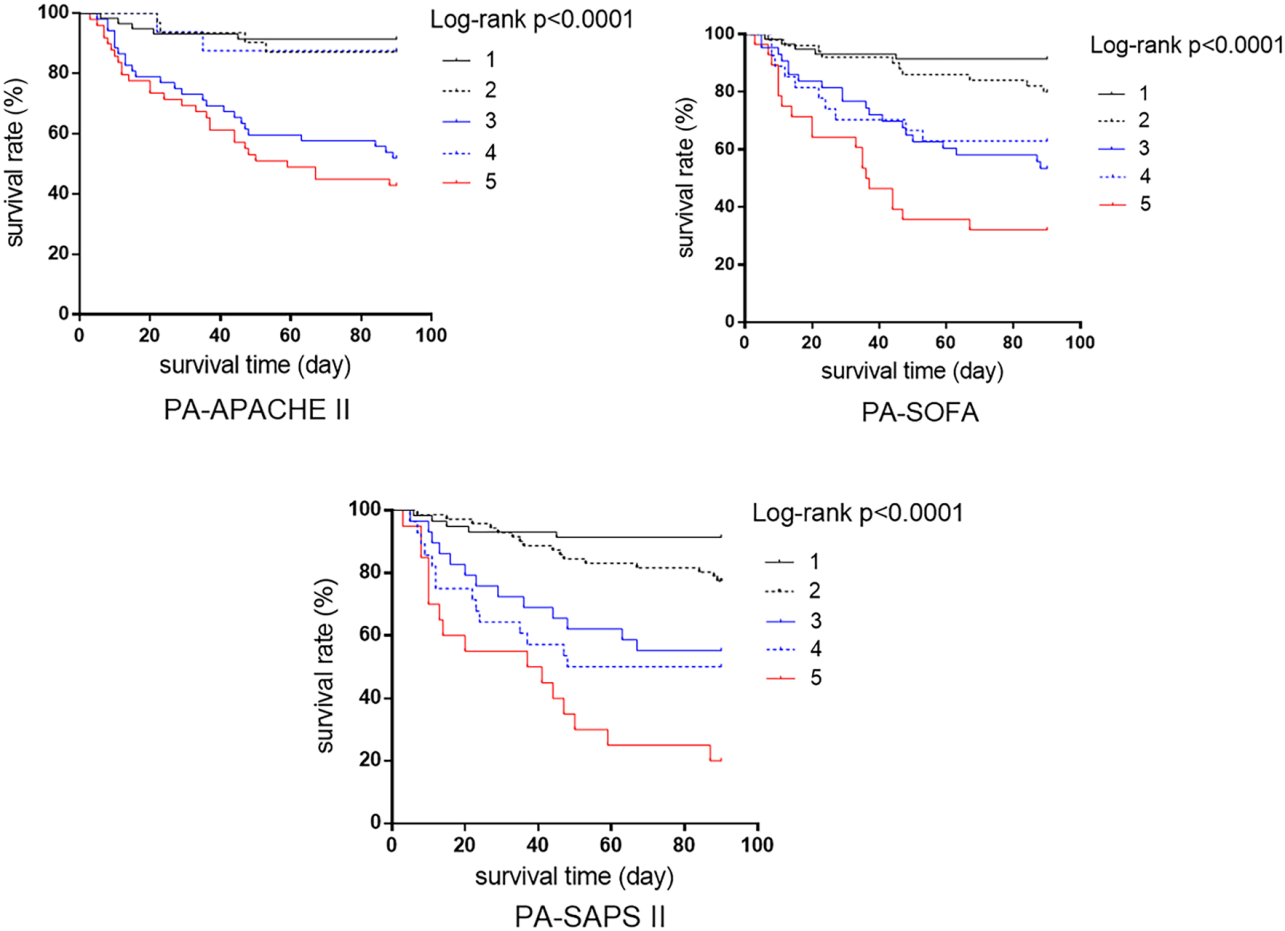
K‒M survival curve of patients according to the combination of the PA value and 3 severity score systems. The scoring method was as follows: first, the PA value was checked to a score of G–G “1”; second, the severity was checked to a score of G–G “2”, B–G “3”, G–B “4”, or B–B “5” (Fig. 2).

K‒M survival curves of patients according to the combination of PA and the 3 severity score systems are shown in Fig. 3. Significant differences in cumulative survival rates among patients with different scores (*P* < 0.05) were found for the 3 combinations. Logistic regression univariate analysis revealed the risk factors for 90-day mortality, including age; baseline Hb level; CRP, PLT, total bilirubin, PA, and IR; and SAPS II, SOFA, APACHE II, PA–SAPS II, PA–SOFA, and PA–APACHE II scores (*P* ≤ 0.05). Correlation coefficient matrix analysis was used to reduce the multicollinearity between variables before they were included in the multivariate regression model for further analysis. The final results revealed that Hb levels (odds ratio [OR], 0.981; 95% CI, 0.963; 0.998; *P* < 0.05), SOFA scores (OR, 1.141; 1.054; 1.235; *P* < 0.05) and PA–SAPS II scores (OR, 2.132; 95% CI, 1.569; 2.899; *P* < 0.05) were found to be independent risk factors for 90-day mortality (Table 2).

**Table 2:** Logistic regression analysis of predictors of 90-day mortality.

	Univariable			Multi-variables		
	OR	95%CI	*P* value	OR	95%CI	*P* value
Age	1.025	1.007, 1.043	0.006			
Hb	0.975	0.961, 0.990	0.001	0.981	0.963,0.998	0.032
CRP	1.004	1.001,1.008	0.019			
PLT	0.992	0.989, 0.996	< 0.001			
TBIL	1.005	1.000, 1.010	0.043			
PA	0.732	0.573, 0.935	0.013			
IR	0.362	0.194, 0.676	0.001			
SAPS II	1.059	1.033, 1.086	< 0.001			
SOFA	1.183	1.103,1.268	< 0.001	1.141	1.054,1.235	0.001
APACHE II	1.109	1.055, 1.166	< 0.001			
PA–SAPS II	2.305	1.758, 3.022	< 0.001	2.132	1.569, 2.899	< 0.001
PA–SOFA	2.002	1.564, 2.564	< 0.001			
PA–APACHE II	1.789	1.432, 2.234	< 0.001			

Hb, hemoglobin; PLT, platelets; TBIL, total bilirubin; CRP, C-reactive protein; PA, phase angle; IR, impedance ratio; APACHE II, acute physiology and chronic health evaluation II; SAPS II, simplified acute physiology score II; SOFA, sequential organ failure assessment; PA–SAPS II, PA–SOFA, and PA–APACHE II are the scores obtained by combining phase angle with SAPS II, SOFA and APACHE II, respectively.

### The association between outcome and scores obtained by combining PA with the SAPS II, SOFA and APACHE II scores

**Figure 4:**
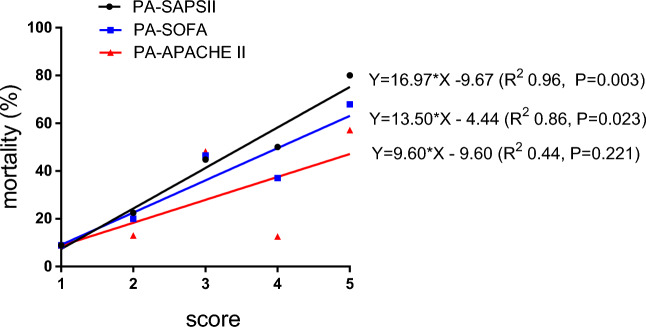
Score-mortality scatter plot. The scoring method was as follows: first, the PA value was checked to obtain a score of G–G “1”; second, the severity score was checked to obtain scores of G–G “2”, B–G “3”, G–B “4”, and B–B “5”. The severity score systems used included the simplified acute physiology score II (SAPS II), sequential organ failure assessment (SOFA), and acute physiology and chronic health evaluation II (APACHE II).

The PA–SAPS II and PA–SOFA scores were linearly associated with 90-day mortality, while the PA–APACHE II score was not. The prediction equations used were as follows: PA–SAPS II, $Y(\%)=16.97\times X-9.67$ (R^2^ = 0.96, *P* = 0.003); PA–SOFA, $Y(\%)=13.50\times X-4.44$ (R^2^ = 0.86, *P* = 0.023); and PA–APACHE II, $Y(\%)=9.60\times X-9.60$ (R^2^ = 0.44, *P* = 0.221) (Fig. 4). A higher R^2^ of the PA–SAPS II suggested that the PA–SAPS II is more valuable than the PA–SOFA score for evaluating disease severity.

## Discussion

The prediction of outcomes in critically ill patients was usually based on the severity scores at baseline or at a specific time point. This study investigated the change trend of severity scores from admission to the 3rd day in the ICU and the association with patient outcomes. On this basis, we used PA, a BIA parameter, to integrate into the severity score change trend and found that the PA–SAPS II score was strongly linearly correlated with 90-day mortality and was an independent risk factor.

As reported in a study on patients with sepsis, a ΔSOFA score between 1 and 7 days was a good predictor of 28-day mortality, with an area under the receiver operating characteristic (AUROC) of 0.812, but was not strongly associated with mortality (R^2^ = 0.315)^20^. Another study on sepsis showed that a change in the SOFA score on the 7th day was a useful prognostic marker of 28-day mortality, and a decrease in the SOFA score at admission of less than 25% was associated with increased mortality (OR 14.87)^8^. Several other studies have suggested that the dynamic assessment of severity scores is a better predictor of patient prognosis^7,9,10,21^. As in our study, we investigated the association between the change in severity score from baseline and outcome, with the assumption that a baseline score of bad or good and a change trend toward bad or good are both important factors affecting outcome. As revealed by the results, patients with the same change trend in severity score but different baselines had quite different outcomes, which indicates that focusing only on the change trend regardless of baseline may weaken the prediction accuracy.

The mortality of patients with very low scores according to current systems still reaches a certain level; for example, the mortality of patients determined as the mildest according to changes in the SAPS II or SOFA score reached 15.8% and 13.3%, respectively, which implies that the disease severity of the non-survivors is not reflected by the score. In contrast, for surviving patients, the severity score may overestimate mortality, as reported by a European multicenter study in the intensive care unit^22^. Theoretically, as an indicator of the reactance properties of the body, which are affected by body composition, tissue edema, and cell damage, the PA value reflects the health status of the body in another dimension, which is quite different from score systems. It has been reported that PA is related to nutrition and prognosis of patients, while the cutoff values were heterogeneous due to different groups and diseases. A recent study reported that PA was associated with sarcopenic obesity in post-stroke patients, and the cutoff values that could predict sarcopenic obesity were 4.29° for men and 3.84° for women^23^. Another study on critically ill patients showed that low PA (< 4.6°) was an independent predictor of 1-year mortality (OR: 1.81; *P* = 0.02) after ICU admission^24^. In this study, patients with PA value greater than 3.7° for the first and third days were found to have a lower mortality rate, which implies its superiority in predicting outcomes in patients with less severe conditions than other scoring systems. As PA is highly sensitive for predicting patients with low mortality and has low sensitivity for predicting patients with high mortality, while the critical illness score has the opposite effect, in this study, we combined the PA value with the change trend of severity scores and found that the PA–SAPS II score had a very strong linear correlation with mortality, which implies that it is an indicator of sensitivity to changes in disease severity.

The present study has obvious limitations. First, the PA–SAPS II grouping method should be validated in another group of critically ill patients. Second, the results from a single-center retrospective analysis with a small sample size still need further verification through studies with larger sample sizes and multicenter prospective investigations. Thirdly, a comparison should be made for the prediction power of delta phase angle and the traditional scores. Finally, the sample size should be expanded to further refine the grouping to obtain more precise scores with more sensitive detection of disease progression.

## Conclusion

The dynamic changes in PA value and severity score were better to predict patients with different outcomes, and the score combining PA and SAPS II score showed a strong linear correlation with 90-day mortality.

## Data Availability

The datasets used and analyzed during the current study are available from the corresponding author on reasonable request.

## References

[1] le Gall JR, Lemeshow S, Saulnier F (1993). A new simplified acute physiology score (SAPS II) based on a European/North American multicenter study. JAMA.

[2] Knaus WA, Draper EA, Wagner DP, Zimmerman JE (1985). APACHE II: A severity of disease classification system. Crit. Care Med..

[3] Lambden S, Laterre PF, Levy MM, Francois B (2019). The SOFA score-development, utility and challenges of accurate assessment in clinical trials. Crit. Care.

[4] Barlow P (2012). A practical review of the Glasgow Coma Scale and score. Surgeon.

[5] Kądziołka I, Świstek R, Borowska K, Tyszecki P, Serednicki W (2019). Validation of APACHE II and SAPS II scales at the intensive care unit along with assessment of SOFA scale at the admission as an isolated risk of death predictor. Anaesthesiol. Intensive Ther..

[6] Rahmatinejad Z (2020). Prognostic utilization of models based on the APACHE II, APACHE IV, and SAPS II scores for predicting in-hospital mortality in emergency department. Am. J. Emerg. Med..

[7] Raith EP (2017). Prognostic accuracy of the SOFA score, SIRS criteria, and qSOFA score for in-hospital mortality among adults with suspected infection admitted to the intensive care unit. JAMA.

[8] Karakike E, Kyriazopoulou E, Tsangaris I, Routsi C, Vincent JL, Giamarellos-Bourboulis EJ (2019). The early change of SOFA score as a prognostic marker of 28-day sepsis mortality: Analysis through a derivation and a validation cohort. Crit. Care.

[9] Moreno R (1999). The use of maximum SOFA score to quantify organ dysfunction/failure in intensive care. Results of a prospective, multicentre study. Working group on sepsis related problems of the ESICM. Intensive Care Med..

[10] Lee H (2015). Efficacy of the APACHE II score at ICU discharge in predicting post-ICU mortality and ICU readmission in critically ill surgical patients. Anaesth. Intensive Care.

[11] Godinjak A (2016). Predictive value of SAPS II and APACHE II scoring systems for patient outcome in a medical intensive care unit. Acta Med. Acad..

[12] Yao J, Zhou M, Xu B, Li C, Chen H, Gong D (2020). The association of bioimpedance analysis parameters with the outcomes of critically ill patients. Clin. Nutr..

[13] Kyle UG (2004). Bioelectrical impedance analysis–part I: Review of principles and methods. Clin. Nutr..

[14] Ward LC, Brantlov S (2023). Bioimpedance basics and phase angle fundamentals. Rev. Endocr. Metab. Disord..

[15] Beberashvili I (2014). Bioimpedance phase angle predicts muscle function, quality of life and clinical outcome in maintenance hemodialysis patients. Eur. J. Clin. Nutr..

[16] Ceolin J, de Borba EL, Mundstock E, de Oliveira JR, Mattiello R, Bodanese LC (2023). Phase angle of bioimpedance as a marker of inflammation in cardiovascular diseases: A systematic review. Nutrition.

[17] Kwon YE (2023). Impact of sarcopenia and phase angle on mortality of the very elderly. J. Cachexia Sarcopenia Muscle.

[18] Nescolarde L, Talluri A, Yanguas J, Lukaski H (2023). Phase angle in localized bioimpedance measurements to assess and monitor muscle injury. Rev. Endocr. Metab. Disord..

[19] Di Vincenzo O, Marra M, Di Gregorio A, Pasanisi F, Scalfi L (2021). Bioelectrical impedance analysis (BIA) -derived phase angle in sarcopenia: A systematic review. Clin. Nutr..

[20] Iba T, Arakawa M, Mochizuki K, Nishida O, Wada H, Levy JH (2019). Usefulness of measuring changes in SOFA score for the prediction of 28-day mortality in patients with sepsis-associated disseminated intravascular coagulation. Clin. Appl. Thromb. Hemost..

[21] Jones AE, Trzeciak S, Kline JA (2009). The sequential organ failure assessment score for predicting outcome in patients with severe sepsis and evidence of hypoperfusion at the time of emergency department presentation. Crit. Care Med..

[22] Poncet A, Perneger TV, Merlani P, Capuzzo M, Combescure C (2017). Determinants of the calibration of SAPS II and SAPS 3 mortality scores in intensive care: A European multicenter study. Crit. Care.

[23] Yoshimura Y (2023). Phase angle is associated with sarcopenic obesity in post-stroke patients. Clin. Nutr..

[24] Stellingwerf F, Beumeler LFE, Rijnhart-de Jong H, Boerma EC, Buter H (2022). The predictive value of phase angle on long-term outcome after ICU admission. Clin. Nutr..

